# Retracted: Stimulatory Effects of Gamma Irradiation on Phytochemical Properties, Mitotic Behaviour, and Nutritional Composition of Sainfoin (*Onobrychis viciifolia* Scop.)

**DOI:** 10.1155/2019/3295627

**Published:** 2019-08-29

**Authors:** The Scientific World Journal

The Scientific World Journal has retracted the article titled “Stimulatory Effects of Gamma Irradiation on Phytochemical Properties, Mitotic Behaviour, and Nutritional Composition of Sainfoin (Onobrychis viciifolia Scop.)” [1]. As raised on PubPeer, several figures also appear in the authors' publications in Journal of Agricultural Science and Technology [2] and Journal of Animal & Plant Sciences [3] and additional concerns with figures and tables were found in comparing the article to the first author's thesis [4], as listed below. The authors' responses to these ethical concerns (https://www.hindawi.com/journals/tswj/ethics/) were not considered adequate by the Editorial Board and therefore the article is being retracted. The first author maintains that the results in the article are correct, but he says the university has restricted the download of the thesis.

Details of the main concerns are as follows:

1. Figure 5(e) (Gamma radiation, fragmented chromosomes) in The Scientific World Journal is identical to Figure 5(f) (Laggard chromosomes) in Journal of Agricultural Science and Technology and Figure 4.36i (UV, Laggard and fragmented chromosomes) in the thesis. The same image of laggard/fragmented chromosomes is used for general cytology (Journal of Agricultural Science and Technology), gamma radiation (The Scientific World Journal), and UV-B (thesis).
2. Table 1 (growth of seeds after gamma irradiation) in The Scientific World Journal is similar to Table 4.35 in the thesis. Growth statistics in The Scientific World Journal and the thesis differ for all but one measure, but many of the digits are the same even when the results differ, e.g. 17.89 ± 2.24 vs 38.89 ± 0.24.
3. Figure 3 (Non-irradiated seeds) in The Scientific World Journal is the same as Figure 4.31a (UV-B exposed) in the thesis, meaning that the HPLC traces for a control in The Scientific World Journal and UVB exposed seeds in the thesis are identical. However, the areas reported are different: 7009 and 9421 vs 8523 and 8825.
4. Table 4 (mitosis) in The Scientific World Journal is not the same as Table 4.38 (mitosis) in the thesis, but shares many digits. When data sets share mainly nonsignificant digits (after the decimal point), this is unlikely to be due to chance.

Additional concerns are as follows:

(5) Figure 3(b) (non-irradiated seeds) in The Scientific World Journal is identical to Figure 2(b) (control seeds) in Journal of Animal & Plant Sciences. The control was reused across different experiments, on gamma radiation and humidity, respectively.
(6) As noted above, Figure 5(e) (Gamma radiation, fragmented chromosomes) in The Scientific World Journal is identical to Figure 5(f) (Laggard chromosomes) in Journal of Agricultural Science and Technology and Figure 4.36i (UV, Laggard and fragmented chromosomes) in the thesis. However, the scales are different (10 *μ*m in TSWJ vs 100 *μ*m in Journal of Agricultural Science and Technology and the thesis)and the background above the chromosomes appears to be slightly different between the images (there is a dark stripe missing in Journal of Agricultural Science and Technology and the thesis).
(7) Figure 5(f) (micronucleus) in The Scientific World Journal is identical to Figure 5(g) (micronucleus) in Journal of Agricultural Science and Technology. The same image is used for general cytology (Journal of Agricultural Science and Technology) and gamma radiation (The Scientific World Journal).
(8) Figure 3 (Irradiated seeds, 120 Gy) in The Scientific World Journal is the same as Figure 4.38a (Gamma Radiation, 120 Gy) in the thesis. However, the heights for sanguinarine differ, 6428 vs 7009, and the areas for berberine, 1078 vs 1087.
(9) The control HPLC traces are very different between The Scientific World Journal (Figure 3, Non-irradiated seeds) and the thesis (Figure 4.38b, Control Seeds).
(10) Table 5 (mitosis) in The Scientific World Journal is not the same as Table 4.39 (mitosis) in the thesis. The cell and nuclear area results are very different in The Scientific World Journal and the thesis.
